# Endoscopic assisted microscopic posterior cordotomy for bilateral abductor vocal fold paralysis using radiofrequency versus coblation

**DOI:** 10.1007/s00405-023-08331-z

**Published:** 2023-12-02

**Authors:** Anwar Abdelatty Ibrahim, Ahmad Mahmoud Hamdan, Ahmed Ali Elnaggar

**Affiliations:** 1 Faculty of Medicine, Otorhinolaryngology Department, Menoufia University, Shebin El-Kom, Menoufia, Egypt; 2 Faculty of Medicine, Otorhinolaryngology Department, Tanta University, Tanta, Egypt

**Keywords:** Bilateral abductor paralysis, Coblation, Vocal fold paralysis, Posterior cordotomy, Radiofrequency

## Abstract

**Purpose:**

To assess the outcomes of endoscopic assisted microscopic posterior cordotomy for bilateral abductor vocal fold paralysis (BAVFP) using radiofrequency versus coblation.

**Methods:**

This was a randomized prospective cohort study that carried out on 40 patients with BAVFP who were subjected to endoscopic/assisted microscopic posterior cordotomy. The patients were randomly allocated into two groups: group (A) patients were operated with radiofrequency, and group (B) patients were operated with coblation. Glottic chink, grade of dyspnea, voice handicap index 10 (VHI10), and aspiration were evaluated pre-operatively and 2 weeks and 3 months post-operatively.

**Results:**

There was a significant improvement in the glottic chink and VHI10 scores postoperatively with a non-significant difference between both groups regarding the degree of improvement. In addition, there was a significant improvement of the grade of dyspnea with a non-significant impact on the degree of aspiration in both groups post operatively. There was a lower incidence of oedema and granulation formation in the coblation group but without a statistical significance.

**Conclusion:**

Both techniques are effective alternatives for performing posterior transverse cordotomy in cases of BAVFP.

## Introduction

The management of bilateral abductor vocal fold paralysis (BAVFP) might be difficult. Establishing a patent airway, maintaining the glottic sphincter’s functionality, and, if possible, maintaining a passable voice quality are the objectives of treating individuals with BAVFP [1]. In patients with symptomatic airway impairment, surgery is essential. It is crucial to conduct a thorough history taking and examination before undergoing surgery to ascertain the cause, gauge the severity of the bilateral mobility limitation, and predict the chance of a spontaneous recovery [2].

There are numerous treatment options that can be used to treat BAVFP. Tracheostomy is well acknowledged and suitable for iatrogenic damage where there is a chance of spontaneous recovery. Endoscopic suture lateralization is another choice. Vocal cordotomy with or without medial partial arytenoidectomy to widen the glottic airway may be pursued when recovery of vocal cord motion is improbable or impossible [3]. The present endoscopic procedures frequently use laser technology to accomplish the cordotomy and partially obliterate the medial aspect of the arytenoid cartilage. These techniques aim to balance expanding the airway with voice preservation and are frequently well tolerated [3]. Laser can be a useful tool for various treatments, however, intraoperative hemorrhage, postoperative granulation tissue, and collateral heat injury are also potential side effects of these procedures [4]. Technology that shortens the operative time and reduces heat-related collateral soft tissue damage is required. The aim of this study was to assess the outcomes of endoscopic assisted microscopic posterior cordotomy for bilateral abductor vocal fold paralysis using radiofrequency versus coblation techniques regarding breathing, voice, and swallowing. The study focused on these two techniques being more available and less expensive when compared with Laser surgery.

## Methods

The current study was a randomized prospective comparative study conducted on 40 patients with BAVFP who were subjected to endoscopic/assisted microscopic posterior cordotomy from 2019 to 2023 after approval of the institutional review board. Informed written consent was taken from every patient before participation in the study.

Participants in the trial had to have moderate to severe stridor at least 12 months after a diagnosis of bilateral abductor vocal fold paralysis with a glottic chink of 3 mm or smaller. The study excluded patients with insufficient cardiopulmonary reserve, unilateral vocal fold paralysis, laryngeal masses, age under 20, persistent chronic aspiration, or unfitness for surgery.

The patients of the study were alternatively allocated into two equal groups. Group A included 20 patients treated with radiofrequency. Seven of them were tracheostomized preoperatively. Group B included 20 patients treated with controlled coblation using electrosurgical ablation probe. Six patients were tracheostomized. Patients of both groups were subjected to the following protocol.

The three authors of the study worked at two different centers with one author at one center using radiofrequency and the other two authors at another center using coblation. Both teams used the same approach (Kashima’s procedure) for posterior cordotomy despite the difference in the used device.

### Preoperative assessment

A thorough history taking, general examination, ENT examination, and flexible laryngoscopy were all part of the pre-operative assessment routine. Prior to surgery, patients were evaluated for their level of dyspnea, which was divided into four categories: none, mild dyspnea (when there was no restriction on daily activity), moderate dyspnea (when there was a mild restriction on daily activity), severe dyspnea (with stridor), and very severe dyspnea (when there was respiratory difficulty requiring tracheostomy). Patients were assessed by the modified Arabic voice handicap index 10 (VHI-10) questionnaire [5] for the assessment of voice and 8 point penetration aspiration scale [6] for assessment of aspiration.

### Surgical technique (Kashima’s procedure)

After the cricoarytenoid joints were palpated intraoperatively, the optimal side for cordotomy was decided upon based on the side with the least range of motion. Surgeon preference was considered if there was no difference between the two sides. Under general anesthesia, the procedure was conducted, and either a tracheostomy tube or a small endotracheal tube (5 or 5.5 microlaryngeal tube) was used to maintain the airway.

The glottic gap was opened with the use of a Kleinsasser laryngoscope. At the back of the membranous vocal fold, a transverse incision was created. With the frequency set to 4 MHz and the passive electrode applied to the shoulder region, the incision in group (A) was made using an Ellman Radiosurgical Instrument adjusted in the partially rectified mode (50 percent cut, 50 percent coagulation), with the frequency setting. A specially designed needle electrode was used to make the incision. An Arthro Care ENT Coblator was used to make the incision in group (B) to resect soft tissue. Both cauterization and coblation were employed as settings. The suggested settings for a laryngeal wand were Cauterization—3 (non-plasma setting) and Coblation—7 (plasma setting). PROcise LW was employed by the coblation wand. The shaft could be bent. It was equipped with a screen electrode that could quickly debulk the target tissue. Its flexible shaft conformed to the anatomy of the patient.

The vocal ligament and the muscle fibers of the thyroarytenoid muscle were completely divided, and the incision continued laterally until it reached the inner perichondrium of the thyroid cartilage. The incision started anterior to the vocal process of the arytenoid cartilage without exposing the cartilage. Since the electrode moved slowly, the cutting was accomplished by the electrode’s energy effect rather than the surgeon’s physical strength. If bleeding occurred, it was stopped by suction diathermy and bipolar laryngeal cautery or by exerting pressure with gauze soaked with adrenaline (1:1000).

### Postoperative management

All patients received dexamethasone intravenously throughout the procedure to lessen laryngeal edema. To reduce fibrosis, this was repeated twice within 48 h of the surgery. This was then followed by a 5-day course of oral steroids (prednisolone), which was then decreased over the following 5-day period. Strong anti-reflux drugs were also administered for 15 days following surgery. After a weaning test, patients who had a tracheostomy were decannulated.

### Postoperative assessment

During the follow-up periods, an endoscopic examination was performed to check for any complications (such as edema, granulations development). The glottic chink, grade of dyspnea, and presence of aspiration were evaluated as early as two weeks after the subsidence of postoperative oedema to evaluate the outcomes of the operation and the feasibility of decannulation in tracheostomized patients. Voice rehabilitation needed a prolonged period of follow up with assessment at 2 weeks and 3 months postoperative.

### Statistical analysis

Statistical analysis was done by SPSS 25 (IBM Corp., Armonk, NY, USA). Quantitative variables turned to be non-normally distributed using Kolmogorov–Smirnov. Quantitative data were presented as mean, standard deviation (SD) and range. Preoperative and postoperative data were compared using Wilcoxon Sign Rank test. While the quantitative data of both groups were compared using Mann–Whitney *U* test. Qualitative variables were expressed as number and percentage and were compared using Chi square test. *P* value less than 0.05 was considered statistically significant. While *p* value less than 0.001 was considered highly significant.

## Results

**Fig. 1:**
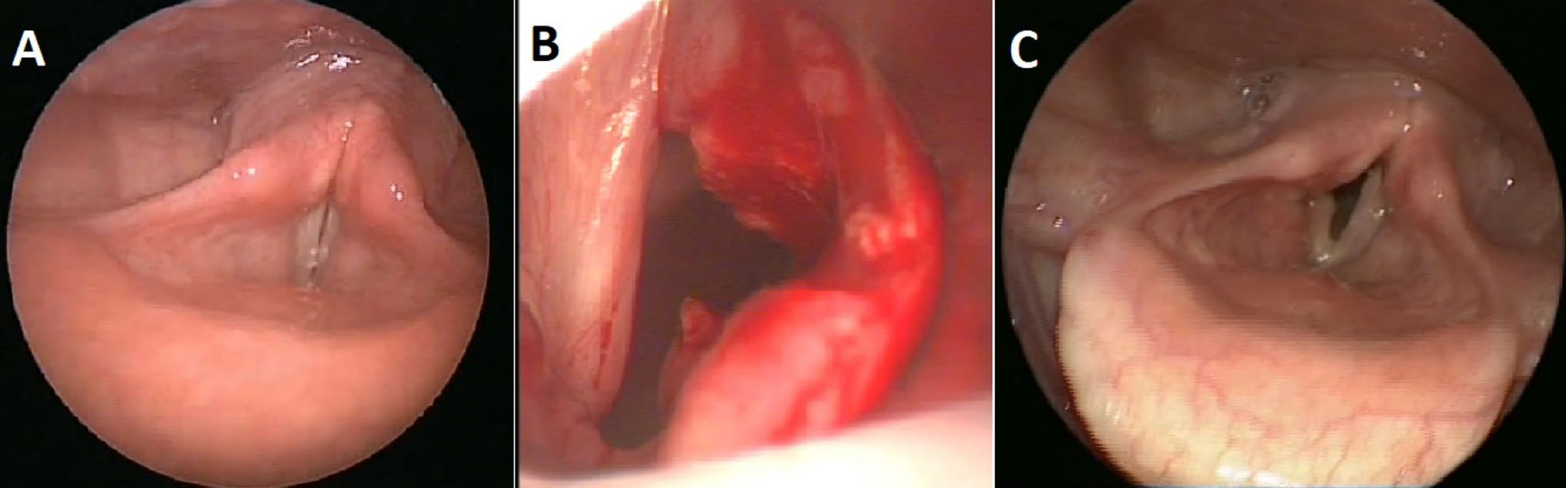
A 43-year-old female patient with bilateral vocal fold paralysis with **A** Preoperative indirect laryngoscopy view showing bilaterally paralyzed vocal cords standing in paramedian position, **B** Intraoperative direct laryngoscopy view showing right posterior cordotomy using radiofrequency, **C** A three-month postoperative indirect laryngoscopy view showing healed cordotomy site with adequate respiratory chink

**Fig. 2:**
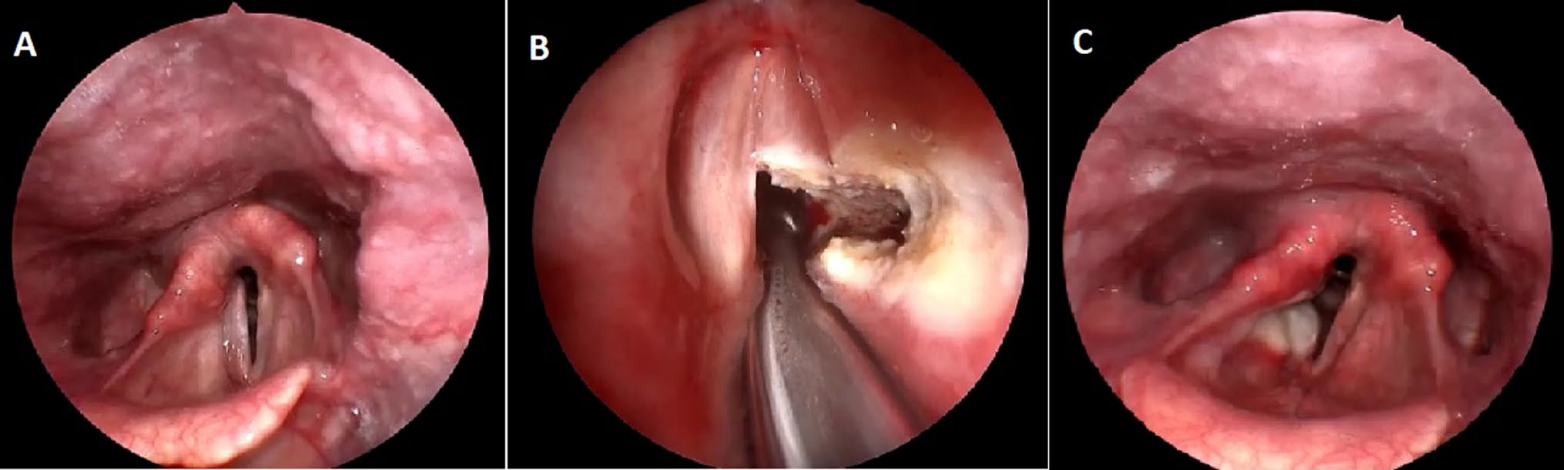
A 36-year-old female patient with bilateral vocal fold paralysis with **A** Preoperative indirect laryngoscopy view showing bilaterally paralyzed vocal cords standing in paramedian position, **B** Intraoperative direct laryngoscopy view showing right posterior cordotomy using coblation, **C** A three-month postoperative indirect laryngoscopy view showing healed cordotomy site with adequate respiratory chink

Group A included 5 (25%) males and 15 (75%) females with ages ranging from 29 to 60 years old (Mean ± SD 41.55 ± 9.55) while Group B included 4 (20%) males and 16 (80%) females with ages ranging from 30 to 61 years old (Mean ± SD 44.9 ± 10.71). There was a non-significant difference between both groups regarding age and sex (*p* = 0.267 and 0.064, respectively). There was a non-significant difference between both groups regarding the preoperative VDI 10 score, grade of dyspnea, and degree of aspiration (*p* = 0.992, 0.73, and 0.705, respectively). The cause of paralysis was idiopathic in 3 (15%) cases in group A and 2 (10%) in group B, while thyroidectomy was the cause for paralysis in the remaining cases of both groups (Table 1). Group A patients was managed with radiofrequency posterior cordotomy (Fig. 1), while group B patients were managed with coblation posterior cordotomy (Fig. 2).

**Table 1:** Comparison between the two studied groups regarding sociodemographic and baseline clinical data

Parameter	Group I	Group II	Statistical test	*p* value
	Mean ± SD Range	Mean ± SD Range	Mann Whitney U test	
Age (years)	41.55 ± 9.55	44.9 ± 10.71	z = 1.10905	0.267
	29–60	30–62		
Preoperative glottic chink	2.43 ± 0.44	2.8 ± 0.62	z = – 1.85293	0.064
	2–3	2–4		
Preoperative VHI 10 score	23.05 ± 1.43	23.45 ± 1.85	z = 0.01353	0.992
		21–27		

	No. (%)	No. (%)	Chi square test	*P* value
*Gender*				
Male	5 (25)	4 (20)	0.1434	0.704
Female	15 (75)	16 (80)		
*Cause of paralysis*				
Idiopathic	3 (15)	2 (10)	Fisher exact test	1
Thyroidectomy	17 (85)	18 (90)		
*Dyspnea*				
Very severe	7 (35)	6 (30)	0.6296	0.73
Severe	10 (50)	9 (45)		
Moderate	3 (15)	5 (25)		
*8-point penetration aspiration scale*				
None	16 (80)	15 (75)	0.1434	0.705
Penetration	4 (20)	5 (25)		
Aspiration	0	0		

*VHI* voice handicap index

There was a significant difference in both study groups comparing preoperative with postoperative glottic chink (*p* = 0.00008 for both). There was a non-significant difference between both groups regarding the degree of change in glottic chink (*p* = 0.928) (Table 2).

**Table 2:** Comparison between the two studied groups according to glottic chink pre & postoperatively:

Group	Preoperative glottic chink Range Mean ± SD	Postoperative glottic chink Range Mean ± SD	Wilcoxon sign rank test	*P* value
Group I (20)	2–3 2.43 ± 0.44	3–5 3.95 ± 0.67	z = – 3.9199	0.00008
Group II (20)	2–4 2.8 ± 0.62	3–5 4.3 ± 0.57	z = – 3.9199	0.00008

Group	Change in glottic chink Range Mean ± SD	Mann Whitney *U* test	*P* value
Group I (20)	1–2.5	Z = 0.09468	0.928
	1.55 ± 0.43		
Group II (20)	1–2		
	1.5 ± 0.32		

There was a significant difference in both study groups comparing preoperative with postoperative modified Arabic VHI10 Egyptian mid Delta accent at 2 weeks and 3 months (*p* = 0.00008 and 0.00014, respectively for each group). There was a non-significant difference between both groups regarding the degree of change in VHI10 (*p* = 0.992) (Table 3).

**Table 3:** Comparison between the two studied groups according to VHI10 preoperatively, 2 weeks and 3 months postoperatively

Group	Preoperative VHI10 Range Mean ± SD	Postoperative VHI10 At 2 weeks Range Mean ± SD	Postoperative VHI10 At 3 months Range Mean ± SD	Wilcoxon -Sign Rank test *P* value at 2 weeks	Wilcoxon -Sign Rank test *P* value at 3 months
Group I (20)	21–26 23.05 ± 1.43	25–36 30 ± 3.88	21–34 26.5 ± 4.17	0.00008	0.00014
Group II (20)	21–27 23.45 ± 1.85	25–36 30.25 ± 4.09	21–35 26.8 ± 4.38	0.00008	0.00014

Group	Change in VHI10 at 2 weeks Mean ± SD	Mann–Whitney *U* test *P* value at 2 weeks	Change in VHI10 at 3 months Mean ± SD	Mann–Whitney *U* test *P* value at 3 months
Group I (20)	3–12 6.95 ± 2.74	0.912	0–11 3.45 ± 3.25	0.992
Group II (20)	4–12 6.8 ± 2.55		0–10 3.35 ± 3.03	

*VHI* voice handicap index

There was a significant improvement in the grade of dyspnea in both groups 2 weeks postoperatively (*p* = 0.0002 and 0.0035, respectively). However, there was a non significant difference between preoperative and postoperative degree of aspiration in both groups (= 0.168 and 0.1025, respectively). Although the radiofrequency grouped showed a higher incidence of postoperative oedema and granulations at 2 weeks postoperative compared with the coblation group, it did not reach a statistical significance (*p* = 0.167 and 0.212, respectively) (Table 4). All tracheostomized patients were successfully decannulated at 3 months postoperatively. No revision surgery was needed in either of the two groups for 3 months follow up period of the study.

**Table 4:** Comparison between Preoperative and postoperative grade of dyspnea and degree of aspiration in both groups at 2 weeks post operative

Parameter	Preoperative	2 weeks postoperative	Statistical test	*P* value
*Group I*				
*Dyspnea*				
None	0	4	22.141	0.0002
Mild	0	7		
Moderate	3	6		
Severe	10	2		
Very severe	7	1		
Aspiration				
None	16	12	1.9048	0.168
Penetration	4	8		
Aspiration	0	0		
*Group II*				
*Dyspnea*				
None	0	4	15.647	0.0035
Mild	0	6		
Moderate	5	6		
Severe	9	3		
Very severe	6	1		
*Aspiration*				
None	15	10	2.6667	0.1025
Penetration	5	10		
Aspiration	0	0		
*Oedema*				
Present	8	4	1.9048	0.167
Absent	12	16		
*Granulations*				
Present	5	2	1.5584	0.212
Absent	15	18		

## Discussion

Bilateral abductor vocal cord paralysis has been treated with a variety of exterior and endoscopic surgical techniques. Each method compares to the others and has certain benefits or drawbacks. An adequate airway is created via endoscopic laser transverse cordotomy or posterior cordectomy, resulting in a voice that is generally of acceptable quality. However, laser operations need specialized equipment and a skilled surgical team. In the current study, there was a significant improvement in the glottic chink and VHI 10 score postoperatively with a non-significant difference between both groups using radiofrequency and coblation regarding the degree of improvement. In addition, there was a significant improvement of the grade of dyspnea with a non-significant impact on the degree of penetration/aspiration in both groups post operatively. In patients with bilateral vocal cord paralysis, Oysu et al. [7] assessed the outcomes of endoscopic posterior cordotomy employing microdissection electrodes, radiofrequency, and an Arrowtip monopolar needle. Evaluation of exercise tolerance, airway, and voice in relation to preoperative and postoperative outcomes revealed that all patients had appropriate functional airways and had good exercise tolerance as opposed to poor preoperative exercise tolerance. The Voice Handicap Index readings before and after surgery did not differ significantly (*P* > 0.05), though. According to Basterra et al. [8], compared to CO2 laser technology, the use of microelectrodes and radiofrequency devices allowed for angled cutting with excellent hemostasis and positive results in terms of shorter surgical times, easier manipulation, and lower costs. Pnarbaşl et al. [9] used a novel technique in which image pixel counts were used to assess the rima glottidis opening of the glottic area postoperatively to assess the efficiency of unilateral posterior cordotomy with radiofrequency in nine patients with bilateral abductor vocal cord paralysis. At the 2-month postoperative visit, video-laryngo-stroboscopic (VLS) images showed an increase in the rima glottidis region opening of approximately 97% (*p* = 0.008).

When contrast to laser, radiofrequency provides the advantage of tactile feedback and does not need safety measures. It is a safe and straightforward operation with better hemostasis, a shorter overall surgical time, and simpler handling than laser. A further life-threatening problem with laser surgery is the possibility of airway fire. The use of an ultrafine needle and a procedure energy level that is insufficient to ignite a spark in the lower airways are two potential explanations for this [8]. In less developed nations, the instrument’s cost-effectiveness becomes more significant. The radiofrequency generator can be used as a bipolar or a monopolar cautery, and the tips are reusable. The biggest drawback of radiofrequency is the initial edema; however, this condition subsides over the course of two months, making tracheostomized patients a better candidate for this operation.

Sethi et al. [10] conducted a retrospective evaluation with fourteen consecutive patients to assess the efficacy and safety of posterior cordectomy utilizing coblation technology. According to postoperative subjective improvements in respiratory distress (in two non-tracheostomized patients) and uneventful decannulation (in 12 tracheostomized patients), there was a considerable improvement in the airway. The postoperative VHI scores did not, however, differ statistically significantly from the preoperative evaluation. Due to a recurrence of respiratory difficulty, one patient needed the surgery to be repeated. There were no unfavorable events recorded during any of the surgeries.

The current study reported a lower incidence of oedema and granulation formation in the coblation group compared with the radiofrequency group but not reaching a statistical significance. Other studies highlighted the benefits of coblation in microlaryngeal surgeries which included minimal or negligible damage to surrounding normal tissue, rapid healing of the mucosal surface of the vocal folds as demonstrated by the return of normal mucosal wave pattern within 6 weeks following surgery, and no risk of airway fire. The negligible bleeding during surgery and minimal formation of exudate decreases the risk of web formation when managing bilateral vocal fold lesions in the same sitting. Other advantages included a reduced risk of infection, and a shorter recovery. With a coblation posterior cordotomy, the voice and other defensive functions of the larynx are preserved, and the precise tissue ablation prevents collateral injury to nearby tissue and allows for early decannulation with no oedema in the tissues surrounding the larynx [10]. The high cost of the coblation wand, which can only be used once because secondary infections and secondary bleeding after coblation surgery using a previously used wand are common, is one of the drawbacks of coblation cordotomy. Reusing wands should also be discouraged because plasma generation is not at its best when wands are reused [11].

The limitations of our study included the subjective assessment of the study parameters other than the glottic chink which was measured objectively as detected in indirect laryngoscopy. To overcome this limitation, all the study patients were assessed by the same investigator preoperatively and postoperatively to minimize the inter-rater variability, and the bias introduced by this subjective assessment. Another limitation was the presence of preoperative tracheostomy in some patients. To overcome this limitation, we used a fenestrated tracheostomy tube in tracheostomized patients which could be closed temporarily during assessment of the study parameters to minimize the bias introduced by the presence of tracheostomy tube.

In conclusion, selection for a treatment for any patient with BAVFP represents a compromise between airway, voice quality and laryngeal competence. Despite new techniques, a perfect treatment BAVFP is still under debate. Both study techniques could be used as effective alternative for performing posterior transverse cordotomy. Both techniques restore sufficient glottic space without causing damage to phonatory and sphincteric functions of larynx.

## Data Availability

Data are available upon request.
